# Exercise mitigates Alzheimer’s disease by targeting ferroptosis driven by cellular senescence

**DOI:** 10.3389/fcell.2025.1742209

**Published:** 2026-01-08

**Authors:** Yuehan Yu, Kang Chen

**Affiliations:** 1 Physical, Aesthetic and Labor Education Centre, Zhejiang Shuren University, Hangzhou, China; 2 Tianjin Key Laboratory of Exercise Physiology and Sports Medicine, Tianjin University of Sport, Tianjin, China

**Keywords:** Alzheimer’s disease, cellular senescence, exercise, ferroptosis, lipid peroxidation

## Abstract

Ferroptosis, a regulated form of cell death driven by iron-dependent lipid peroxidation, has emerged as a critical link between cellular senescence and Alzheimer’s disease (AD). Senescent cells disrupt iron metabolism, promote peroxidation-prone lipid remodeling, and suppress antioxidant defenses, creating a pro-ferroptotic environment that accelerates neuronal degeneration. This review integrates recent mechanistic evidence demonstrating that these senescence-induced changes heighten ferroptotic susceptibility and drive AD pathology through pathways involving protein aggregation, autophagic failure, and inflammatory synaptic loss. Importantly, physical exercise has emerged as a pleiotropic intervention that counteracts these ferroptotic mechanisms at multiple levels. Exercise restores iron homeostasis, reprograms lipid metabolism to reduce peroxidation risk, reactivates antioxidant systems such as GPX4, enhances mitochondrial and autophagic function, and suppresses chronic neuroinflammation. Moreover, systemic adaptations through muscle, liver, and gut axes coordinate peripheral support for brain health. By targeting ferroptosis driven by cellular senescence, exercise not only halts downstream neurodegenerative cascades but also interrupts key upstream drivers of AD progression. These findings position ferroptosis as a therapeutic checkpoint linking aging biology to neurodegeneration and establish exercise as a mechanistically grounded strategy for AD prevention and intervention.

## 1 Introduction

The pathological heterogeneity of Alzheimer’s disease (AD) necessitates a conceptual shift beyond the conventional amyloid and tau centric framework (Stockwell, 2022; Majernikova et al., 2024; Sun et al., 2023). As the leading cause of age-related dementia, AD imposes an immense global health and socioeconomic burden (Stern, 2012). Aging represents the predominant risk factor for AD, driving a continuum of physiological and molecular alterations that collectively predispose the brain to neurodegeneration. These include progressive mitochondrial dysfunction (Swerdlow, 2018), chronic oxidative and metabolic stress (Liu et al., 2022a), persistent neuroinflammation (Leng and Edison, 2021), and impaired proteostasis, all of which synergistically compromise neuronal resilience and accelerate the development of canonical pathological hallmarks such as amyloid-beta (Aβ) plaque deposition and hyperphosphorylated tau aggregation (Jamal et al., 2025; Long and Holtzman, 2019). Recent studies further indicate that age related iron dyshomeostasis and redox disequilibrium are integral components of these degenerative processes, contributing to excessive oxidative damage and increased cellular vulnerability in both neurons and glial cells (Yan et al., 2021; Chen et al., 2025a). Because these age dependent perturbations converge on iron metabolism, lipid oxidation, and antioxidant defense pathways, increasing attention has been directed toward ferroptosis, an iron dependent form of regulated cell death that may represent a critical mechanistic bridge linking aging with AD pathology.

Ferroptosis has emerged as a distinct and tightly regulated form of cell death driven by iron catalyzed lipid peroxidation and failure of antioxidant defenses (Tang et al., 2021). Unlike apoptosis, necroptosis, or autophagy, ferroptosis is characterized by Glutathione peroxidase 4 (GPX4) inactivation, accumulation of lipid hydroperoxides, and dysregulated iron metabolism that collectively culminate in oxidative membrane damage (Liu et al., 2022). Postmortem analyses of AD model and transgenic animal models consistently reveal signatures of ferroptotic stress, including reduced GPX4 expression, depletion of glutathione, and increased levels of ferrous iron within hippocampal and cortical regions (Wang et al., 2022a; Bao et al., 2021). These biochemical and histopathological findings indicate that ferroptosis contributes to the cascade of neuronal loss and synaptic dysfunction characteristic of AD (Bao et al., 2021). Mechanistically, iron dyshomeostasis amplifies Fenton chemistry, generating hydroxyl radicals that promote polyunsaturated phospholipid peroxidation and mitochondrial injury. In parallel, defective antioxidant systems such as the System Xc-–glutathione–GPX4 axis and auxiliary pathways involving ferroptosis suppressor protein 1 (FSP1) and dihydroorotate dehydrogenase (DHODH) fail to neutralize lipid peroxides, thereby exacerbating oxidative damage (Stockwell et al., 2017; Li et al., 2022; Tan et al., 2025). Moreover, the release of lipid peroxidation products and damage associated molecular patterns activates glial cells and triggers neuroinflammatory responses, forming a vicious cycle that accelerates amyloid deposition, tau pathology, and network degeneration (Perluigi et al., 2024; Zhu et al., 2022). Collectively, these findings establish ferroptosis not as a secondary consequence but as a key pathogenic amplifier within the oxidative metabolic network that drives AD progression.

Emerging evidence supports exercise as a systemic intervention that mitigates ferroptosis by targeting the upstream disruptions and downstream consequences of aging related stress (Chen et al., 2023a; Xu et al., 2025a; Huang et al., 2024). Physical activity restores iron homeostasis by reducing systemic iron overload (Peinado et al., 2021), normalizing hepatic hepcidin levels (Liu et al., 2023b), and enhancing ferroportin expression in both peripheral tissues and the brain. In aged models, exercise downregulates transferrin receptor 1 and divalent metal transporter 1, limiting non transferrin bound iron accumulation and mitochondrial iron overload (Schumacher et al., 2002; Fernandez-Real et al., 2009). Concurrently, exercise suppresses ferroptosis prone lipid remodeling by attenuating the expression of acyl CoA synthetase long chain family member 4 and lipoxygenases, thereby reducing phospholipid peroxidation risk (Huang et al., 2024; Burgomaster et al., 2008). It also preserves antioxidant capacity by maintaining glutathione levels, upregulating GPX4, and activating ferroptosis suppressor protein 1, dihydroorotate dehydrogenase, and related protective pathways (Wang et al., 2024a; Li et al., 2024). Mitochondrial integrity and autophagic flux are restored, while the senescence associated secretory phenotype and neuroinflammation are suppressed (Pahlavani, 2023; Yang et al., 2022; Hu et al., 2024a; Zhang et al., 2025a). At the systems level, exercise coordinates anti ferroptotic responses across the liver brain, gut brain, and immune axes, enhancing clearance of oxidized metabolites and promoting a neuroprotective environment (Yu and Chen, 2025; Song et al., 2025a; Islam et al., 2021). Collectively, these multiscale effects position exercise as a pleiotropic modulator capable of counteracting senescence driven ferroptotic cascades in the aging brain.

Here, we elucidate how cellular senescence increases susceptibility to ferroptosis, how ferroptosis contributes to AD progression, and how exercise mitigates these deleterious processes through multilevel regulation of iron metabolism, lipid peroxidation, and antioxidant defense. By integrating evidence from molecular, cellular, and systemic studies, this review establishes ferroptosis as a critical convergence point linking age related metabolic dysfunction with neurodegenerative pathology. Moreover, we highlight exercise as a potent physiological intervention capable of reprogramming senescence associated ferroptotic pathways, thereby interrupting the self-amplifying oxidative and inflammatory cascade and preserving neuronal and cognitive integrity. Collectively, this integrative perspective provides a conceptual framework for understanding the interplay among aging, ferroptosis, and exercise, offering new insights into therapeutic strategies for delaying or preventing AD.

## 2 Cellular senescence as a driver of ferroptosis susceptibility in AD

**FIGURE 1:**
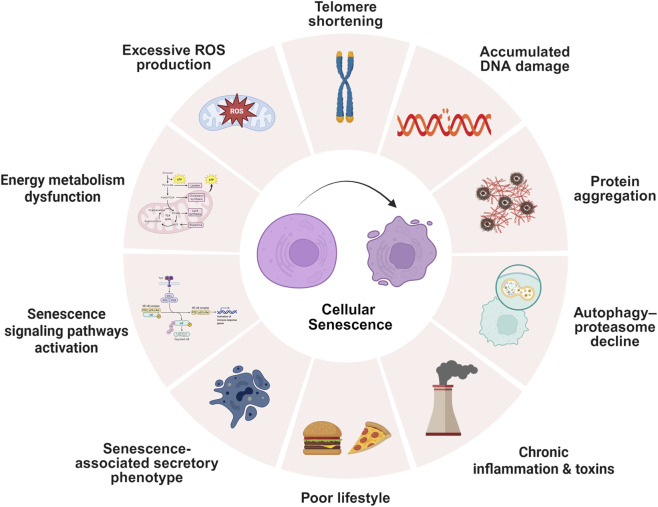
Cellular senescence triggers and associated mechanisms.

**FIGURE 2:**
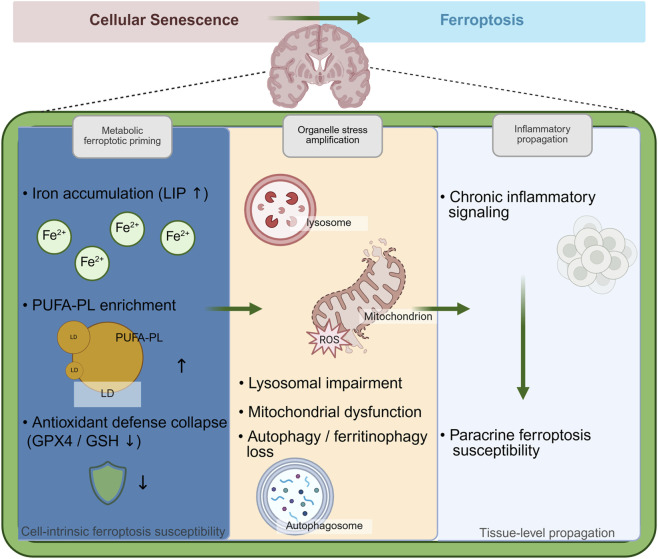
Cellular senescence programs ferroptosis susceptibility in AD.

Cellular senescence represents a state of permanent growth arrest triggered by various stressors, including DNA damage, oxidative stress, shortened telomeres, and oncogenic signals (Figure 1) (Ajoolabady et al., 2025; Zhang et al., 2024a; Chen et al., 2007; de Magalhaes and Passos, 2018; Di Micco et al., 2021). While this process initially protects against cancer development (McHugh et al., 2025), the gradual accumulation of senescent cells during aging disrupts normal tissue function. These cells release a complex mixture of inflammatory molecules, growth factors, and tissue-remodeling enzymes collectively termed the senescence-associated secretory phenotype (SASP) that persistently alters the surrounding tissue environment (Huang et al., 2022; Dong et al., 2024). In AD, markers of cellular senescence appear in brain regions critical for memory, including the hippocampus and cortex, before traditional pathological hallmarks or cognitive symptoms emerge (Bussian et al., 2018; Wang et al., 2021; Zhang et al., 2019; Musi et al., 2018; Habib et al., 2020). These markers include the cyclin-dependent kinase inhibitors p16INK4A and p21CIP1, along with increased senescence-associated β-galactosidase (SA-β-gal) activity. Different brain cell types respond distinctly to senescence. Neurons exhibit impaired synaptic function, while glial cells including astrocytes, microglia, and oligodendrocyte progenitor cells adopt proinflammatory phenotypes characterized by sustained SASP activity (Table 1) (Kwong et al., 2012; Le Bars and Glaab, 2025; Pang et al., 2022; Kudo et al., 2024; Gross et al., 2025; Matsudaira et al., 2023). The inflammatory and metabolic changes induced by senescent cells create conditions that render neighboring cells vulnerable to ferroptosis, an iron-dependent form of regulated cell death characterized by lipid peroxidation. This vulnerability arises from multiple converging factors comprising increased oxidative stress, disrupted iron homeostasis, depleted antioxidant reserves, and altered lipid composition (Park et al., 2021; Currais et al., 2025; Thorwald et al., 2025; Liu et al., 2025). Paradoxically, senescent cells themselves often resist ferroptosis through compensatory mechanisms such as lysosomal iron sequestration. However, their persistent presence generates a pro-oxidative milieu that promotes ferroptosis in surrounding non-senescent cells (Feng et al., 2024a; Dasgupta et al., 2024; Wang et al., 2024b). This dual mechanism creates a spreading pattern of ferroptotic that contributes to progressive neurodegeneration. The mechanistic link between senescence and ferroptosis susceptibility involves several interconnected pathways. These include dysregulated iron metabolism, phospholipid remodeling, antioxidant system failure, mitochondrial and lysosomal dysfunction, and persistent SASP-mediated inflammatory signaling (Figure 2) (Tang et al., 2025; Abdukarimov et al., 2025; Tai et al., 2017; Feng et al., 2024b; Khan et al., 2024). These processes act synergistically to establish cellular senescence as a critical upstream regulator of ferroptosis in AD pathogenesis.

**TABLE 1:** Senescence-driven checkpoints of ferroptosis susceptibility.

Model system	Pathway axis	Key molecules	Ferroptosis-relevant outcomes	References
Human diploid fibroblasts (irradiation-induced senescence)	Iron handling	↑ lysosomal Fe^2+^; ↓ ATP6V1C2	↑ labile iron pool; lysosomal iron accumulation	Loo et al. (2025)
Mouse skeletal muscle (OA-induced aging)	Iron handling	↑ Fe^2+^/Fe^3+^; ↑ ACSL4	↑ iron burden; enhanced ferroptotic susceptibility	Xiang et al. (2025)
Human skeletal myoblasts (doxorubicin-induced senescence)	Iron handling	↑ FTH1/FTL; ↓ NCOA4; ↓ FPN	↑ labile iron pool; impaired ferritin turnover	Feng et al. (2024a)
Mouse lung tissue (bleomycin-induced fibrosis)	Iron handling	↑ ferritin; ↑ labile Fe^2+^	↑ iron accumulation; ferroptosis-prone microenvironment	Maus et al. (2023)
Mouse liver tissue (AMLN diet–induced aging)	Antioxidant defenses	↓ GPX4	Compromised lipid peroxide detoxification	Vetuschi et al. (2022)
Vascular smooth muscle cells (aging mouse)	Iron handling	↑ NCOA4; ↑ labile iron	Enhanced ferritinophagy-driven iron release	Li et al. (2023a)
Arterial tissue (aging vascular model)	Iron handling	↑ TfR1; ↓ FPN	↑ tissue iron burden	Sun et al. (2024a)

This circular diagram illustrates the diverse stressors and biological processes contributing to cellular senescence, a state of irreversible cell cycle arrest. Key triggers include telomere shortening, accumulated DNA damage, excessive reactive oxygen species (ROS) production, energy metabolism dysfunction, senescence signaling pathway activation, SASP, autophagy-proteasome decline, protein aggregation, poor lifestyle factors, and chronic inflammation with toxins. These factors collectively disrupt cellular homeostasis, as depicted by the central senescent cells, highlighting the multifaceted nature of senescence in aging and disease contexts.

This schematic illustrates how cellular senescence creates a permissive environment that primes ferroptosis and facilitates its contribution to AD pathology. At the cellular level, senescence induces metabolic ferroptotic priming, characterized by iron accumulation, Polyunsaturated fatty acids-enriched membrane remodeling, and impairment of GPX4/GSH- dependent antioxidant defenses, thereby increasing intrinsic susceptibility to lipid peroxidation. Concurrent failure of organelle quality control—including lysosomal and mitochondrial dysfunction and reduced autophagy/ferritinophagy—amplifies oxidative stress and ferroptotic vulnerability. At the tissue level, senescence-associated inflammatory signaling propagates ferroptosis susceptibility through paracrine mechanisms. Collectively, these processes position cellular senescence as an upstream driver that licenses ferroptosis and accelerates neurodegeneration and cognitive decline in AD.

### 2.1 Core metabolic reprogramming establishes a ferroptosis-prone state

Cellular senescence induces a coordinated metabolic reprogramming that converges on iron dyshomeostasis, peroxidation-prone lipid remodeling, and collapse of antioxidant defenses, collectively establishing a ferroptosis-prone cellular state in the aging brain (Di Sanzo et al., 2016). A central feature of senescent cells is persistent disruption of iron homeostasis, resulting in sustained expansion of the labile iron pool (Gorska et al., 2023). Senescence is associated with increased expression of iron uptake machinery, including transferrin receptor 1 and divalent metal transporter 1 (Guo et al., 2025), together with hepcidin-mediated degradation of ferroportin, the sole cellular iron exporter. These changes promote intracellular iron retention and enhance redox-active ferrous iron availability, thereby facilitating Fenton chemistry and oxidative stress (Bao et al., 2021; Ma et al., 2025; Cairo and Recalcati, 2007; Slusarczyk et al., 2023). Excess intracellular iron directly reshapes lipid metabolism by promoting the incorporation of polyunsaturated fatty acids into membrane phospholipids (Liang et al., 2022). Senescent cells exhibit upregulation of acyl-CoA synthetase long-chain family member 4 and lysophosphatidylcholine acyltransferase 3, which preferentially channel arachidonic and adrenic acids into phosphatidylethanolamines that are highly susceptible to peroxidation (Feng et al., 2024a; Liang et al., 2022; Doll et al., 2017; Alves et al., 2025). Structural features of senescent cells, including cellular hypertrophy and endoplasmic reticulum expansion, further increase the membrane surface area requiring antioxidant protection, amplifying lipid peroxidation vulnerability. Notably, the ferroptosis-promoting effects of iron-driven lipid remodeling are exacerbated by collapse of lipid antioxidant defense systems. Senescence impairs the cystine–glutathione–GPX4 axis through transcriptional repression and epigenetic silencing of SLC7A11, reduced NADPH availability, and destabilization of GPX4 protein (Liang et al., 2023; Liu et al., 2020). As a result, lipid hydroperoxides accumulate beyond the detoxification capacity of GPX4, rendering senescent cells and their neighbors highly vulnerable to ferroptotic membrane damage (Abdukarimov et al., 2025; Chen et al., 2021; Wang et al., 2020; Saini et al., 2023; Huang et al., 2025; Liu et al., 2017; Yan et al., 2024a; Song et al., 2025b). Together, iron accumulation, peroxidation-prone lipid remodeling, and failure of GPX4-centered antioxidant defenses form a tightly coupled metabolic axis that defines ferroptosis susceptibility in senescent cells. This core axis provides the molecular foundation upon which organelle dysfunction and inflammatory signaling further amplify ferroptotic stress, linking cellular senescence to neurodegenerative vulnerability in AD.

### 2.2 Failure of organelle quality control amplifies ferroptotic stress

Beyond core metabolic reprogramming, senescent cells exhibit progressive failure of organelle quality control systems, which does not initiate ferroptosis but markedly amplifies and sustains ferroptotic stress. Dysfunction of mitochondrial, lysosomal, and autophagic pathways disrupts cellular containment of iron and oxidized lipids (Agrawal et al., 2018; Zhang et al., 2025b; Saimoto et al., 2025), thereby reinforcing the pro-ferroptotic environment established by metabolic rewiring. Impaired ferritinophagy represents a critical failure point linking organelle dysfunction to iron toxicity in senescent cells. Nuclear receptor coactivator 4 (NCOA4), which normally mediates selective autophagic degradation of ferritin, is dysregulated during senescence, leading to inefficient iron recycling and progressive expansion of the cytosolic labile iron pool (Santana-Codina et al., 2021). As lysosomal storage capacity becomes saturated, iron leakage further fuels Fenton chemistry and lipid peroxidation, reinforcing ferroptotic vulnerability. Mitochondrial dysfunction further amplifies ferroptotic stress by enhancing ROS generation and disrupting iron–sulfur cluster homeostasis (Liu et al., 2022). Senescent mitochondria exhibit impaired electron transport chain activity, leading to electron leakage and excessive superoxide production (Fang et al., 2019). These changes promote lipid peroxidation and sensitize mitochondrial membranes to ferroptotic damage, while release of redox-active iron from damaged mitochondria exacerbates cytosolic oxidative stress. Concomitantly, lysosomal dysfunction compromises cellular sequestration of iron and oxidized macromolecules (Liu et al., 2023c). Downregulation of vacuolar H^+^-ATPase subunits impairs lysosomal acidification, resulting in reduced activity of acid-dependent hydrolases and destabilization of lysosomal membranes (Chen et al., 2021; Anandhan et al., 2023). Although transient iron sequestration within lysosomes may initially limit cytosolic oxidative stress, chronic lysosomal alkalinization promotes iron release and sustains lipid peroxidation under senescent conditions. Collectively, breakdown of organelle quality control transforms metabolic ferroptotic susceptibility into persistent cellular stress. By disrupting iron containment, lipid handling, and redox buffering, mitochondrial and lysosomal dysfunctions act as amplifiers that lock senescent cells into a self-reinforcing ferroptotic state. This organelle-level failure provides a critical mechanistic bridge between core metabolic reprogramming and the tissue-wide propagation of ferroptotic stress described below.

### 2.3 SASP acts as a systems-level amplifier of ferroptosis susceptibility

While metabolic rewiring and organelle dysfunction define cell-intrinsic ferroptosis susceptibility, the SASP extends this vulnerability beyond individual cells (Zhang et al., 2022a; Zhang et al., 2024b). Through sustained release of inflammatory cytokines, chemokines, and lipid mediators, senescent cells convert localized ferroptotic stress into a tissue-wide, self-propagating microenvironment that amplifies ferroptosis susceptibility across the aging brain (Wei et al., 2023; Herdy et al., 2022). A primary mechanism by which the SASP amplifies ferroptotic risk is its direct reinforcement of the iron–lipid–antioxidant axis (Zhang et al., 2023; Jiang et al., 2023). Pro-inflammatory cytokines, particularly IL-6, activate STAT3 signaling and induce hepcidin expression, thereby promoting ferroportin degradation and intracellular iron retention (Hirano, 2021; Ye et al., 2023). Concurrently, SASP-driven NF-κB activation enhances expression of lipid-remodeling enzymes such as ACSL4 and lipoxygenases, while suppressing cystine uptake and glutathione synthesis through downregulation of SLC7A11. These changes further intensify iron accumulation, lipid peroxidation, and GPX4 insufficiency within both senescent and neighboring cells.

Beyond reinforcing intracellular metabolic stress, SASP signaling promotes paracrine ferroptosis susceptibility in surrounding neural cells (Zhang et al., 2022a). Persistent exposure to senescence-derived cytokines elevates ROS production, disrupts redox homeostasis, and sensitizes neighboring neurons and glia to lipid peroxidation–induced membrane damage (He et al., 2017; Basisty et al., 2020). This bystander effect allows ferroptotic vulnerability to spread spatially, even in cells that do not exhibit classical senescence markers. At the tissue level, SASP-mediated inflammation establishes a permissive microenvironment that links ferroptosis to subsequent neurodegenerative processes (Saul et al., 2022). Chronic activation of NF-κB signaling sustains cytokine production, alters lipid biosynthesis, and weakens antioxidant defenses, while inflammatory stress primes glial cells toward reactive phenotypes (Wei et al., 2023; Wang et al., 2019). These changes create a feedforward loop in which ferroptotic stress and neuroinflammation mutually reinforce one another, setting the stage for synaptic dysfunction and network instability in AD. Collectively, the SASP functions as a systems-level amplifier that integrates cellular senescence with ferroptosis propagation. By reinforcing metabolic ferroptotic axes, enabling paracrine vulnerability, and sustaining chronic inflammation, SASP signaling transforms senescence-associated stress into a self-perpetuating, tissue-wide ferroptotic state. This transition provides a critical mechanistic link between senescence-driven susceptibility and the emergence of widespread neuronal and synaptic pathology discussed in the following section.

## 3 Ferroptosis promotes AD pathology

**FIGURE 3:**
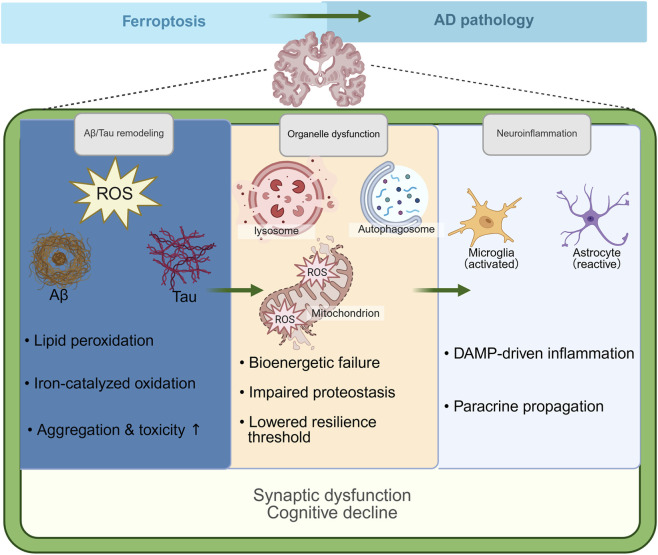
Ferroptosis-driven amplification of AD pathology.

Emerging evidence identifies ferroptosis as a critical amplifier of AD pathology, operating through multiple interconnected mechanisms that converge on neuronal dysfunction and death (Table 2). This cell death pathway interfaces with hallmark AD features through a hierarchical cascade of biochemical and cellular disruptions rather than functioning in isolation. Iron dyshomeostasis establishes a pro-oxidative environment that accelerates both Aβ and tau protein aggregation. Simultaneously, lipid peroxidation generates reactive aldehydes that form toxic adducts with these proteins, reinforcing their misfolding and accumulation (Majernikova et al., 2024; Dahlmanns et al., 2023; Tian et al., 2025; Ayton et al., 2020). This oxidative stress overwhelms the degradative capacity of autophagic and lysosomal systems, resulting in concurrent accumulation of undegraded protein aggregates and redox-active iron species (Tian et al., 2025; Muhoberac and Vidal, 2019). As ferroptotic cells accumulate damage, they release damage-associated molecular patterns (DAMPs) that activate microglia and astrocytes. This activation amplifies neuroinflammatory responses and triggers complement-mediated synaptic pruning (Sharma et al., 2025; Liu et al., 2023d; Goldsteins et al., 2022). These ferroptosis-induced events ultimately culminate in widespread synaptic loss and functional network disruption, which represent key drivers of cognitive impairment in AD. These mechanisms function within a self-reinforcing pathological network where ferroptotic processes at molecular, organellar, and intercellular levels amplify one another. This systems-level integration accelerates the transition from early biochemical stress to overt neurodegeneration (Figure 3), establishing ferroptosis as a mechanistic bridge between upstream metabolic dysregulation and downstream structural and functional brain damage in AD.

**TABLE 2:** How ferroptosis amplifies Alzheimer’s pathology: mechanism–outcome–evidence.

Mechanistic module	Immediate consequence	Downstream seeding/aggregation, pathology, synapse	AD link	Representative readouts/biomarkers	References
Iron export deficiency	↓FPN, ↑Labile iron	↑Lipid peroxidation, Ferroptotic neuronal death	↑Aβ aggregation, ↑tau protein hyperphosphorylation, Synaptic loss	↑MDA, ↓GSH, ↓GPX4, Behavioral memory tests	Bao et al. (2021)
Lipid raft iron-lipid interaction	↑Lipid peroxidation in rafts, ↓Ferroptosis suppressors	↑Neuroinflammation, ↑Neuronal vulnerability	↑Aβ plaques, Cognitive decline	4-HNE, GPX4 activity, Chelation effects in mice	Thorwald et al. (2025)
Amyloid-iron interaction	↑Aβ-iron binding, Ferroptosis activation	↑ROS, Membrane damage	↑Aβ aggregation, Tau modification	Lipid ROS, Iron levels, Immunohistochemistry	Majernikova et al. (2024)
Transcriptomic changes	↑Oxytosis/ferroptosis genes	↑Cellular stress, Death pathways activation	↑Neurodegeneration, Synapse dysfunction	Gene expression profiles, GSH/GSSG, LC3-II	Currais et al. (2025)
Glial cell regulation	↑Glial activation, Ferroptosis propagation	↑Neuroinflammation, Iron retention	↓Aβ clearance, ↑Tau spread	↓GPX4, ↑IL-6, Microglial markers, Electrophysiology	Xu et al. (2025b)
SIRT1-FSP1 signaling	Ferroptosis induction by APP-overexpression, Mitochondrial damage	Learning/memory defects, Neuronal ferroptosis	APP overexpression, Cognitive impairment	SIRT1/FSP1 mRNA/protein expression, Behavioral tests (learning/memory)	Yan et al. (2024b)

This schematic illustrates how ferroptosis amplifies AD pathology across molecular, cellular, and tissue levels. Ferroptosis-associated oxidative stress and lipid peroxidation drive pathological remodeling of Aβ and tau, which in turn exacerbate mitochondrial, lysosomal, and autophagic dysfunction, thereby reducing neuronal resilience. At the tissue level, ferroptotic damage elicits DAMP-driven neuroinflammation and paracrine propagation of vulnerability through glial reprogramming, ultimately converging on synaptic dysfunction and cognitive decline.

### 3.1 Ferroptosis-driven Aβ and Tau pathological remodeling

Ferroptosis acts as a potent pathological amplifier in AD by reshaping the biochemical and redox environment that governs Aβ and tau aggregation (Zha et al., 2025). Rather than functioning solely as a terminal cell death program, ferroptosis generates sustained lipid peroxidation and iron-catalyzed oxidative stress that directly modify the structure, toxicity, and clearance of these hallmark proteins. Lipid peroxidation products, including 4-hydroxynonenal (4-HNE) and malondialdehyde, covalently modify Aβ and tau, promoting oligomerization, fibril formation, and membrane-disruptive properties (Wongjaikam et al., 2025; Tan et al., 2022). Oxidative modification of tau cysteine residues enhances hyperphosphorylation and filament assembly, while peroxidized lipid membranes increase neuronal vulnerability to Aβ-induced toxicity. Experimental models consistently demonstrate that ferroptotic stress accelerates both Aβ accumulation and tau pathological conversion, effects that are mitigated by iron chelation or inhibition of lipid peroxidation (An et al., 2024; Yong et al., 2024; Sun et al., 2024b). Notably, iron plays a permissive yet decisive role in this process. Elevated ferrous iron catalyzes Fenton chemistry, amplifying ROS production and reinforcing lipid peroxidation cascades (Park and Chung, 2019). Iron directly interacts with histidine residues in Aβ and cysteine residues in tau, stabilizing aggregation-prone conformations (Cheignon et al., 2018; Xie et al., 2012). Neuroimaging and postmortem analyses further reveal spatial convergence of iron accumulation with amyloid plaques and neurofibrillary tangles in vulnerable brain regions, supporting a topographic coupling between ferroptotic stress and proteinopathy (Ayton et al., 2020). Moreover, ferroptosis also undermines proteostatic mechanisms that normally constrain Aβ and tau burden. Oxidative damage and lysosomal membrane instability impair autophagic and lysosomal degradation pathways, reducing clearance efficiency and allowing toxic aggregates to persist (Chen et al., 2023b). Together, ferroptosis-driven lipid peroxidation, iron-catalyzed oxidation, and proteostatic failure establish a self-reinforcing loop that accelerates amyloid and tau pathology, thereby priming the neurodegenerative cascade characteristic of AD.

### 3.2 Organelle dysfunction links ferroptosis to neuronal vulnerability

Once ferroptosis is initiated, it does not remain confined to lipid peroxidation at the plasma membrane but rapidly propagates through disruption of organelle homeostasis (Tang et al., 2024; Zhang et al., 2025c), thereby amplifying neuronal vulnerability in AD. Mitochondria and lysosomes—key regulators of energy metabolism and proteostasis—emerge as central targets and effectors of ferroptotic stress (Feng et al., 2024c). Ferroptosis profoundly compromises mitochondrial integrity and function. Iron-catalyzed lipid peroxidation damages mitochondrial membranes, impairs respiratory chain activity, and disrupts ATP production (Guo et al., 2022a), leading to sustained bioenergetic failure. Mitochondrial ROS further amplify lipid peroxidation, reinforcing ferroptotic stress and weakening synaptic maintenance and neuronal resilience (Qin et al., 2021). In this context, mitochondrial dysfunction represents both a consequence and a driver of ferroptosis, forming a feed-forward loop that accelerates neuronal decline. Moreover, lysosomal integrity is similarly undermined during ferroptosis, with critical implications for protein clearance (Chen et al., 2023c). Oxidative damage destabilizes lysosomal membranes and impairs acidification, reducing the activity of cathepsins and other degradative enzymes essential for Aβ and tau turnover (Chen and Zhong, 2014; Dhapola et al., 2024). As a result, autophagic flux becomes ineffective, allowing misfolded and aggregated proteins to accumulate. This failure of lysosome-dependent proteostasis links ferroptotic stress directly to the persistence and expansion of AD–associated proteinopathies. Importantly, ferroptosis-driven organelle dysfunction integrates metabolic stress with impaired clearance pathways. Mitochondrial energy deficits constrain autophagy initiation and vesicular trafficking, while lysosomal dysfunction prevents efficient degradation of damaged organelles and protein aggregates (Peng et al., 2025; Tang et al., 2022). Through this coordinated collapse of organelle quality control, ferroptosis transforms localized oxidative injury into a global cellular vulnerability state, lowering the threshold for synaptic dysfunction and neuronal loss. Together, mitochondrial and lysosomal failure act as a mechanistic bridge connecting ferroptosis to progressive neuronal fragility in AD. By destabilizing energy metabolism and proteostatic capacity, ferroptosis amplifies neurodegenerative processes beyond immediate lipid damage, setting the stage for inflammatory activation and circuit-level dysfunction.

### 3.3 Ferroptosis-induced neuroinflammation and glial reprogramming

Beyond its direct effects on neurons, ferroptosis exerts profound non–cell-autonomous influences by reshaping the inflammatory milieu of the AD brain (Hu et al., 2024b). Ferroptotic cells release DAMPs, oxidized lipids, and iron-rich debris, which act as potent immunogenic signals that activate and reprogram glial populations (Yu et al., 2023; Lei et al., 2025). Microglia are particularly sensitive to ferroptosis-associated cues (Cui et al., 2021). Exposure to lipid peroxidation products and iron overload promotes a shift toward reactive, pro-inflammatory phenotypes characterized by elevated cytokine production and impaired phagocytic efficiency (Lu et al., 2025). This glial reprogramming not only amplifies local oxidative stress but also compromises microglial clearance of Aβ and cellular debris (Sun et al., 2024b), thereby reinforcing protein accumulation and neurotoxicity. In this manner, ferroptosis converts microglia from homeostatic sentinels into drivers of chronic neuroinflammation. Subsequently, astrocytes similarly undergo functional remodeling in response to ferroptotic stress. Oxidative and inflammatory signals derived from ferroptotic cells bias astrocytes toward neurotoxic reactive states, reducing metabolic and trophic support for neurons while enhancing inflammatory signaling (Fan et al., 2024). These changes weaken synaptic maintenance and exacerbate neuronal susceptibility to both ferroptosis and secondary inflammatory injury (Xiong et al., 2025). Crucially, ferroptosis-induced glial activation establishes a feed-forward inflammatory circuit at the tissue level. Activated microglia and astrocytes release cytokines and reactive mediators that further destabilize redox homeostasis, sensitize neighboring cells to lipid peroxidation, and propagate ferroptotic vulnerability across neural networks (Park et al., 2021). Through this mechanism, ferroptosis transcends a cell-intrinsic death pathway and becomes an organizing force for sustained neuroinflammation. ollectively, ferroptosis-driven glial reprogramming links oxidative cell death to chronic inflammatory amplification in AD. By integrating lipid peroxidation, iron dysregulation, and immune activation, ferroptosis transforms localized cellular damage into a tissue-wide pathological process that accelerates neurodegenerative progression.

### 3.4 Systems-level consequences: synaptic failure and cognitive decline

At the systems level, the cumulative effects of ferroptosis-driven proteinopathy, organelle dysfunction, and neuroinflammation converge on synaptic failure and cognitive impairment, the clinical hallmarks of AD (Osse et al., 2022). Synapses are particularly vulnerable to ferroptotic stress due to their high metabolic demand, lipid-rich membranes, and dependence on tightly regulated redox homeostasis (Lish et al., 2025). Lipid peroxidation compromises synaptic membrane integrity and disrupts neurotransmitter release, while mitochondrial dysfunction limits ATP availability required for synaptic maintenance and plasticity (Badimon et al., 2020; Han et al., 2021). Concurrently, chronic ferroptosis-associated inflammation alters synaptic pruning and weakens neurotrophic support, further destabilizing neuronal circuits (Sun et al., 2021a; Hong et al., 2016). These combined insults result in progressive synapse loss and impaired network connectivity, which correlate more closely with cognitive decline than amyloid burden alone. Through these converging mechanisms, ferroptosis functions not merely as a cell death pathway but as a systems-level pathological amplifier that links cellular senescence to circuit dysfunction and cognitive deterioration in AD. By lowering the resilience of synapses and neural networks, ferroptosis accelerates the transition from molecular pathology to functional impairment.

## 4 Exercise ameliorates ferroptosis pathways

Physical activity represents a powerful non-pharmacological intervention for preventing cognitive decline (Yu and Chen, 2025; Galle et al., 2023; Castellote-Caballero et al., 2024). Epidemiological and clinical evidence consistently demonstrates that regular exercise reduces AD risk and delays disease onset (Gaitan et al., 2021; Cezar et al., 2021). Exercise confers direct neuroprotective benefits through modulation of fundamental cellular stress pathways involved in neurodegeneration, extending beyond its established cardiovascular and metabolic effects. Ferroptosis, an iron-dependent cell death mechanism driven by lipid peroxidation, has emerged as a critical convergence point linking age-related metabolic dysfunction, oxidative stress, and chronic neuroinflammation to neuronal loss in AD (Zha et al., 2025; Qiu et al., 2023). Emerging mechanistic studies reveal that exercise counteracts ferroptosis through coordinated biological pathways. Exercise restores iron homeostasis, reprograms lipid metabolism to decrease peroxidation susceptibility, and activates antioxidant defense systems including the glutathione-GPX4 pathway (Hao et al., 2024; Gutierre et al., 2025). Additionally, physical activity enhances mitochondrial and autophagic quality control mechanisms, suppresses the senescence-associated secretory phenotype and neuroinflammation, and coordinates systemic anti-ferroptotic signaling through liver-brain, gut-brain, and immune communication networks (Yang et al., 2022; Ornish et al., 2024; De Miguel et al., 2021). These interconnected mechanisms establish a comprehensive protective network that disrupts ferroptosis and its upstream triggers, providing an integrated approach to preserve neuronal integrity and cognitive function in AD (Table 3).

**TABLE 3:** Exercise interventions to mitigate ferroptosis: pathways, targets, and outcomes.

Study model/population	Exercise modality	Brain region	Key ferroptosis-relevant readouts	Principal findings	References
Older adults (aging cohort)	Habitual physical activity	Hippocampus	Brain iron (QSM), memory	Physical activity attenuates hippocampal iron accumulation and iron–memory coupling	Lee et al. (2023)
Mild cognitive impairment (women)	Aerobic exercise (6-month RCT)	Hippocampus	Hippocampal volume, cognition	Exercise increases hippocampal volume and improves cognitive performance	ten Brinke et al. (2015)
AD patients	Exercise intervention	CSF/plasma–brain axis	Inflammatory markers	Exercise reduces central and peripheral inflammatory burden	Jensen et al. (2019)
APP/PS1 mice	Aerobic treadmill exercise	Prefrontal cortex	Iron overload, lipid peroxidation, GPX4	Exercise mitigates iron-driven lipid peroxidation and restores GPX4	Li et al. (2024)
APP/PS1 mice	Long-term aerobic exercise	Hippocampus	Keap1–Nrf2–GPX4 axis	Exercise enhances ferroptosis defense and improves learning and memory	Wang et al. (2024a)
5xFAD mice	Voluntary exercise	Whole brain	Iron homeostasis	Physical exercise normalizes brain iron metabolism	Belaya et al. (2021)

### 4.1 Exercise restores iron homeostasis and mitigates age-related iron overload

Progressive iron accumulation occurs in the hippocampus, prefrontal cortex, and substantia nigra during aging and AD (Zecca et al., 2004). This excess iron increases neuronal vulnerability to ferroptosis by generating ROS through iron-dependent redox reactions (Park et al., 2021). Physical exercise restores iron homeostasis through coordinated regulation of iron uptake, storage, export, and systemic redistribution pathways (Hao et al., 2024). Aerobic training enhances ferroportin expression, the primary iron exporter, in neurons, astrocytes, and microglia, facilitating cellular iron efflux (Belaya et al., 2021). Concurrently, exercise downregulates transferrin receptor 1, which mediates iron uptake, effectively reducing intracellular iron accumulation (Schumacher et al., 2002). Exercise further suppresses hepcidin, a liver-derived hormone that inhibits ferroportin function (Fensham et al., 2023). This suppression occurs through inhibition of IL-6 and signal transducer and activator of transcription 3 signaling (Badenhorst et al., 2015). Exercise-induced myokines, including IL-10 and IL-15, counteract pro-inflammatory hepcidin induction, thereby enhancing iron export capacity (Antosiewicz et al., 2013).

Neuroimaging studies in animal models and human subjects demonstrate that aerobic exercise reduces iron accumulation in brain regions vulnerable to cognitive decline (Chen and Xiao, 2014; Choi et al., 2021). Exercise decreases iron content in the cortex and hippocampus of AD models (Gutierre et al., 2025). Clinical studies in individuals with mild cognitive impairment show that aerobic training reduces hippocampal iron deposition while improving memory performance (Martinez-Horta et al., 2021). These findings establish that exercise reverses iron overload and promotes functional recovery. In addition, exercise enhances iron storage capacity by upregulating ferritin, the protein complex responsible for safe iron sequestration (Li et al., 2024). This process involves increased expression of molecular chaperones including heat shock proteins and glucose-regulated protein 78 (GRP78), which facilitate proper ferritin subunit assembly (Khadir et al., 2016). These mechanisms ensure that excess iron remains in a redox-inactive form, preventing lipid peroxidation. Exercise also regulates ferritinophagy, the selective degradation of ferritin, through adenosine monophosphate-activated protein kinase signaling (Yang et al., 2023). This regulation maintains iron availability for physiological functions while preventing ferroptosis.

Beyond its effects on brain iron homeostasis, systemic iron also distribution undergoes beneficial modulation through exercise-mediated inter-organ communication. Muscle-derived factors, particularly irisin, stimulate hepatic iron mobilization and enhance iron transport across the BBB (Wei et al., 2020). These adaptations facilitate iron clearance from the central nervous system and reduce iron-mediated oxidative stress. Exercise also alleviates inflammation-associated iron deposition in oligodendrocytes, which require substantial iron for myelin synthesis (Gutierre et al., 2025). In demyelination models relevant to AD, physical training reduces oligodendroglial iron overload and preserves myelin integrity, preventing ferroptosis-associated damage (Mirza et al., 2024). These findings demonstrate that exercise reestablishes iron balance across cellular, tissue, and systemic levels, neutralizing a fundamental driver of ferroptosis and protecting against iron-mediated neurodegeneration in AD.

### 4.2 Exercise remodels lipid oxidation and enhances the GPX4 antioxidant defense

Remodeling of lipid metabolism represents a central mechanism through which exercise reduces ferroptotic vulnerability in AD (Gaitan et al., 2021). Diseased brains are characterized by excessive enrichment of polyunsaturated fatty acids within membrane phospholipids, creating a biochemical environment that favors lipid peroxidation and ferroptotic damage (Lin et al., 2022; Nagpal et al., 2019). Exercise counteracts this pro-ferroptotic state by limiting the availability of oxidizable lipid substrates and reinforcing cellular capacity to detoxify lipid peroxides. At the level of lipid substrates, aerobic training suppresses pathways that promote polyunsaturated fatty acid incorporation into membrane phospholipids, thereby reducing the pool of peroxidation-prone lipids (Huang et al., 2024; Shahtout et al., 2024). This shift in membrane composition lowers susceptibility to lipid radical propagation and enhances cellular resistance to ferroptotic stress. Consistent with these changes, exercise reduces lipid ROS accumulation and preserves metabolic stability in neurons exposed to ferroptosis-inducing conditions (Huang et al., 2024). Moreover, exercise also rebalances lipid handling in glial cells, where dysregulated lipid storage and turnover can amplify oxidative stress (Haney et al., 2024; Prakash et al., 2025). By promoting lipid utilization and reducing oxidized lipid burden, training shifts the cerebral lipid milieu toward a less inflammatory and less ferroptosis-permissive state (Gaitan et al., 2021; Maak et al., 2021). Lipidomic studies support this reprogramming, revealing a decline in pro-oxidant lipid species alongside enrichment of lipid mediators associated with resolution of inflammation and tissue protection (Jensen et al., 2019; Morais et al., 2025). These lipid profile changes correlate with improved structural integrity and cognitive performance in experimental models and clinical cohorts (Kavanagh et al., 2019).

Beyond substrate remodeling, exercise enhances antioxidant defenses that are essential for neutralizing residual lipid peroxides (Ionescu-Tucker and Cotman, 2021). Training restores glutathione availability and increases the activity of lipid peroxide–detoxifying systems, thereby reinforcing a critical barrier against ferroptotic injury (Li et al., 2024). This antioxidant reinforcement is particularly relevant in aging, where endogenous redox buffering capacity progressively declines (Wang et al., 2023). In parallel, exercise engages complementary ferroptosis-suppressing pathways that further stabilize membrane redox homeostasis and limit lipid radical chain reactions (Bersuker et al., 2019; Fanet et al., 2021). Together, exercise confers resistance to ferroptosis by simultaneously reducing lipid peroxidation pressure and strengthening antioxidant detoxification capacity. This coordinated metabolic reprogramming provides a robust and system-level defense against ferroptotic stress in AD, exceeding the protective potential of single-target interventions.

### 4.3 Exercise suppresses SASP and neuroinflammation, disrupting ferroptosis amplification loops

Chronic neuroinflammation creates a permissive microenvironment for ferroptosis in AD (Zhang et al., 2024c; Chirillo et al., 2020). Senescent glial cells secrete SASP factors, including IL-1α, IL-1β, IL-6, TNF-α, and various chemokines (Wei et al., 2023; Gaikwad et al., 2021). These factors recruit peripheral immune cells and induce neuronal ferroptosis. The cytokines upregulate pro-ferroptotic enzymes such as ALOX15 and ALOX12 while suppressing GPX4 expression through STAT3-dependent transcriptional repression (Ren et al., 2024a; Miao et al., 2025). This inflammaging phenotype correlates with hippocampal iron accumulation and cognitive deterioration in AD cohorts.

Physical exercise, particularly moderate-intensity aerobic training, markedly reduces both systemic and central SASP levels. In transgenic AD models, exercise decreases pro-inflammatory cytokine expression, diminishes amyloid burden, and improves spatial learning (Chen et al., 2025b). Human studies have yielded consistent findings. Aerobic interventions in individuals with mild cognitive impairment attenuate serum SASP markers and decrease neuroinflammation, as evidenced by reduced translocator protein binding and preservation of gray matter volume on neuroimaging (Mielke et al., 2025). These anti-inflammatory effects are mediated in part by exercise-induced exerkines such as irisin, which activates PPARγ and FGF21 signaling to suppress SASP, and β-hydroxybutyrate, which inhibits assembly of the NLRP3 inflammasome (Tu et al., 2023). Exercise also attenuates NF-κB signaling, a central driver of SASP transcription (Okudan et al., 2025). Training stabilizes IκBα and reduces p65 phosphorylation while simultaneously activating PPARγ and SIRT1, both of which repress inflammatory gene expression through RelA/p65 deacetylation (Zhao et al., 2023). These molecular changes produce broad suppression of inflammatory cytokines in the hippocampus. Clinical evidence supports these findings. Exercise correlates with reduced IL-6 and TNF-α in serum, alongside lower levels of neuroinflammatory biomarkers such as YKL-40 in cerebrospinal fluid and increased sTREM2, indicative of improved microglial phagocytic function (Mygind et al., 2016). Notably, individuals carrying the APOE4 allele, who are more susceptible to chronic inflammation, demonstrate enhanced benefit through exercise-induced disruption of lipid raft organization and inhibition of interleukin-6 and STAT3 signaling (Corlier et al., 2018).

Microglial phenotype remodeling represents another essential mechanism. In the AD brain, M1-polarized microglia exacerbate ferroptosis by promoting lipid peroxidation and iron retention (Zhou et al., 2025). Exercise drives a shift toward the M2 anti-inflammatory phenotype, increasing IL-10 and TGF-β expression while enhancing phagocytosis via STAT6 and IL-4 signaling (Lu et al., 2017). TREM2 and scavenger receptors such as CD36 and SR-A are upregulated, supporting Aβ clearance and lipid efflux (Zhang et al., 2022b). M2 microglia also produce nitric oxide, which chelates ferrous iron, thereby reducing the labile iron pool and limiting Fenton reaction-driven oxidative damage (Guo et al., 2022b). Human brain organoid models recapitulate these changes (Majernikova et al., 2024). Treatment with exercise-mimetic lactate enhances M2 polarization and mitigates Aβ-induced ferroptosis via the TREM2 and Syk pathway (Zhang et al., 2021a; Jiang et al., 2025). Exercise modulates SASP by targeting key transcriptional regulators. CEBPβ nuclear accumulation is inhibited through SIRT1-mediated deacetylation, reducing the secretion of pro-inflammatory mediators such as IL-8, CXCL1, and MMP-9 (Chen et al., 2024). Concurrently, STAT3 phosphorylation is suppressed through SOCS3 induction, disrupting IL-6 autocrine loops (Jiang et al., 2021). Beyond its senomorphic effects, exercise also exhibits senolytic activity by promoting autophagic clearance of senescent cells through TFEB activation and increased caspase-3-dependent apoptosis (Abokyi et al., 2023). This dual action reduces the senescent cell burden and dampens paracrine ferroptosis signals. The connection between inflammation and ferroptosis is further reinforced through iron metabolic dysregulation. IL-6 upregulates hepcidin via the JAK and STAT3 axis, promoting iron sequestration by increasing transferrin receptor expression and degrading ferroportin, thereby expanding the labile iron pool (Faradina et al., 2023). In response to these pathological changes, exercise reverses this cascade by suppressing inflammatory signaling and restoring iron export capacity. Moreover, exercise preserves the antioxidant glutathione-GPX4 system through Nrf2 and HO-1 activation, which effectively mitigates lipid peroxidation and interrupts the vicious cycle of SASP, ferroptosis, and inflammaging. Notably, in transgenic AD models, exercise decouples IL-6-hepcidin signaling, enhances ferroportin-mediated iron efflux, and reduces hippocampal iron accumulation as well as lipid peroxide formation, collectively providing a crucial mechanism for protection against ferroptosis.

### 4.4 Exercise enhances mitochondrial function and autophagy systems, restoring cellular quality control

Mitochondrial dysfunction and autophagy impairment are major contributors to ferroptosis vulnerability in the aging brain (Adeniyi et al., 2023). Damaged mitochondria enriched with iron-sulfur clusters generate ROS through Fenton chemistry and release mitochondrial DAMPs that exacerbate neuroinflammation. Exercise restores mitochondrial quality control mechanisms, thereby preventing the accumulation of ferroptosis-prone organelles (Wang et al., 2022b; Gleitze et al., 2021). One key pathway involves the stabilization of PINK1 and recruitment of Parkin, both of which are upregulated following moderate aerobic training (Sliter et al., 2018). In transgenic AD models, exercise promotes the accumulation of PINK1 on depolarized mitochondria and facilitates Parkin translocation through phospho-ubiquitin signaling (Kang et al., 2025). This process initiates the clearance of dysfunctional mitochondria, preventing further release of labile iron and mitochondrial ROS. Ubiquitination of mitochondrial outer membrane proteins, including MFN1, MFN2, and VDAC1, is enhanced in exercised animals, accompanied by p62-mediated recruitment to LC3-positive phagophores (Chen et al., 2022). Subsequent lysosomal degradation is evidenced by colocalization with LAMP1 and is associated with reduced mitochondrial oxidative stress and improved cognitive performance (Zhang et al., 2021b). In parallel, exercise activates receptor-mediated mitophagy pathways independent of PINK1 and Parkin. Expression of BNIP3 and NIX increases through HIF1α stabilization, enhancing mitophagy via LC3-interacting region motifs and promoting turnover of dysfunctional mitochondria (Ehrlicher et al., 2020). Additional mitophagy receptors, including FUNDC1 and AMBRA1, are also engaged through ULK1 activation and BCL2 dissociation, respectively (Ma et al., 2023). Exercise further restores cardiolipin externalization on mitochondrial membranes, facilitating direct LC3 binding and mitophagic clearance in neuronal cells exposed to Aβ (McCoin et al., 1985; Chen et al., 2025c). The transcription factor TFEB acts as a master regulator of exercise-induced autophagy enhancement. Exercise activates TFEB through the AMPK and SIRT1 signaling axis, leading to its nuclear translocation and transcriptional activation of genes involved in autophagy, mitophagy, and lysosomal biogenesis (Morais et al., 2023; Huang et al., 2019). TFEB binds E-box motifs on promoters of ATG9B, LAMP1, LAMP2, and CTSD, and enhances V-ATPase expression, restoring lysosomal acidification essential for autophagosome degradation and iron recycling (Abokyi et al., 2023; Matthews et al., 2023). In AD models, TFEB activation mimics the benefits of exercise, reducing lysosomal alkalinization, increasing autophagic flux, and suppressing ferroptosis in primary neurons (Gu et al., 2022). Clinical studies corroborate these findings. Exercise elevates circulating TFEB levels and improves markers of lysosomal function while decreasing cerebrospinal fluid levels of lipid peroxidation byproducts (Andrade-Souza et al., 2020).

Selective autophagy pathways provide additional layers of protection against ferroptosis. Ferritinophagy, mediated by NCOA4, is modulated by exercise through AMPK-dependent phosphorylation, limiting excessive iron release from ferritin stores (Qin et al., 2021; Wu et al., 2023). Lipophagy is similarly enhanced, with SIRT1-mediated deacetylation of ATG7 and ULK1 promoting LC3 recruitment to PLIN-coated lipid droplets, thereby reducing polyunsaturated fatty acid availability for lipid peroxidation (Sun et al., 2021b). Exercise also supports chaperone-mediated autophagy, restoring LAMP2A oligomerization and enhancing the degradation of iron-bound protein aggregates such as pathological tau (Zhuang et al., 2025). The AMPK, SIRT1, and TFEB axis orchestrates these multi-level quality control systems by promoting NAD^+^ biosynthesis through NAMPT, thereby fueling SIRT1 activity and mitigating mTORC1 hyperactivation. Sustained exercise maintains this axis in the hippocampus of aged animals, enhancing overall autophagic efficiency and improving cognitive function (Morais et al., 2025). The temporal dimension of exercise training is notable. Shorter regimens preferentially activate acute clearance pathways such as PINK1 and Parkin, whereas extended interventions promote long-term TFEB-mediated biogenesis and organelle turnover. Together, these mechanisms confer robust resistance to ferroptotic stress.

### 4.5 Exercise modulates systemic metabolism and immunity to counteract ferroptosis in the brain

Exercise elicits systemic adaptations that extend beyond skeletal muscle to orchestrate protective inter-organ communication, ultimately enhancing the brain’s resistance to ferroptosis. Peripheral dysfunctions such as hepatic iron overload, gut microbial imbalance, and chronic inflammation propagate pro-ferroptotic signals through circulating mediators and neurovascular interactions (Santos et al., 2020; Peng et al., 2021; Kuziak et al., 2025). By integrating endocrine, metabolic, and exosomal signaling across multiple organ and brain axes, physical activity reconfigures systemic iron, lipid, and antioxidant homeostasis to suppress ferroptosis initiation and amplification in the central nervous system (Majernikova et al., 2024; Mezzanotte and Stanga, 2024).

Among these protective mechanisms, the liver and brain axis is a key conduit of this systemic protection. Exercise elevates circulating levels of GPLD1, a hepatokine that crosses the BBB and cleaves glycosylphosphatidylinositol-anchored neuronal surface proteins, thereby stabilizing TrkB receptors and enhancing BDNF signaling in the hippocampus (Horowitz et al., 2020). This cascade promotes adult neurogenesis and synaptic plasticity while reinforcing GPX4-mediated ferroptosis defense. Concurrently, exercise also improves hepatic metabolic efficiency, reducing the peripheral iron load and enhancing export via ferroportin while attenuating hepcidin synthesis through inhibition of the IL-6 and STAT3 pathway (Song et al., 2025a; Wang et al., 2025). These hepatic adjustments collectively limit brain iron accumulation and suppress expression of lipid peroxidation enzymes such as ACSL4 in the hippocampus. Complementing these hepatic contributions, muscle-derived myokines further strengthen neuroprotection. Irisin, cleaved from FNDC5 in response to PGC1α activation, exerts pleiotropic effects by promoting neuronal survival through the PI3K, Akt, and ERK1/2 pathways while suppressing neuroinflammation via NF-κB inhibition (Kim et al., 2025). Irisin also enhances mitochondrial metabolism and stabilizes ferroptosis defense mechanisms (Cutuli et al., 2023), including GPX4 and FSP1 with CoQ10 signaling. Moreover, exercise-induced exosomal miR-484, derived from skeletal muscle, crosses the BBB and directly downregulates ACSL4 expression, rebalancing iron and lipid interactions (Huang et al., 2024). In AD models, the synergistic co-activation of irisin and miR-484 significantly mitigates Aβ-induced lipid peroxidation, preserves synaptic integrity, and improves cognitive outcomes (Huang et al., 2024). In addition to liver and muscle contributions, the gut and brain axis also plays a crucial role in this protective network. Aerobic exercise reshapes the gut microbiota by increasing microbial diversity and enriching short-chain fatty acid-producing taxa, including Coprococcus, Eubacterium, and Faecalibacterium (Luo et al., 2024). The resulting elevation of butyrate and other metabolites modulates microglial activity through GPR41 and GPR43 receptors and enhances the antioxidant response by activating the Nrf2 and HO-1 pathway (Hu et al., 2024a). Supporting this mechanism, fecal microbiota transplantation from exercised donors restores short-chain fatty acid levels and ameliorates Aβ pathology and behavioral deficits in transgenic mice (Li et al., 2023b). Furthermore, exercise also modulates the kynurenine pathway by reducing pro-inflammatory metabolites such as quinolinic acid while increasing the production of neuroprotective kynurenic acid (Lu et al., 2024), thereby stabilizing GPX4 and dampening excitotoxic stress associated with ferroptosis. These organ-specific adaptations are further reinforced by systemic iron regulation is improvements through exercise-mediated suppression of hepcidin and promotion of erythropoiesis, limiting iron transfer to the brain. Hepatic upregulation of LRP1 and IDE facilitates peripheral Aβ clearance across the BBB (Lin et al., 2024; Ide and Secher, 2000). Additionally, changes in bile acid metabolism induced by exercise-associated microbes enhance secondary bile acid synthesis, which in turn activates FXR and TGR5 pathways in hepatocytes to suppress NF-κB and reduce hepatic hepcidin production (Kakde et al., 2024). Working synergistically with these metabolic changes, the immune system contributes to the overall protective response. Exercise reprograms peripheral immune cells, promoting anti-inflammatory M2 polarization of monocytes and macrophages while enhancing microglial phagocytic function through TREM2 activation (Zhang et al., 2022b). Furthermore, liver and muscle-derived GDF15, upregulated in response to physical training (Flaherty et al., 2025), acts via the GFRAL receptor to suppress systemic inflammation and ferroptotic signaling. Importantly, these system-level adaptations unfold in a time-dependent manner. Short-term training primarily triggers acute molecular responses such as increased GPLD1 and irisin secretion, as well as early microbiome remodeling (Ren et al., 2024b). In contrast, sustained exercise over longer durations consolidates these effects and activates broader immunometabolic networks and epigenetic reprogramming. Consistent with these temporal dynamics, population-based studies reveal that whole-body aerobic exercise confers more robust protection against cognitive decline than localized interventions, underscoring the essential role of coordinated peripheral and central communication in suppressing ferroptosis and maintaining neurocognitive health.

### 4.6 Translational synthesis and exercise prescription

Based on converging evidence from animal models and human studies, exercise emerges as a robust, multi-target intervention capable of suppressing ferroptosis through coordinated regulation of iron metabolism, lipid peroxidation, antioxidant defense, and neuroinflammation (Table 4). Importantly, these protective effects display clear dependence on exercise modality, intensity, and duration, enabling formulation of mechanistically informed exercise prescriptions. Aerobic exercise represents the most consistently supported modality for ferroptosis suppression (Li et al., 2024). Moderate-intensity continuous training preferentially restores iron homeostasis, remodels ferroptosis-prone lipid substrates, and enhances antioxidant capacity, whereas higher-intensity aerobic exercise further amplifies mitochondrial and redox adaptations. Resistance training alone shows more variable effects on ferroptosis-related pathways but may complement aerobic exercise by improving systemic metabolic resilience. Across species, intervention duration emerges as a critical determinant of efficacy. Short-term exercise primarily induces acute antioxidant and metabolic responses (Zhang et al., 2024d), while sustained training over weeks to months consolidates ferroptosis resistance through structural remodeling, improved organelle quality control, and durable anti-inflammatory reprogramming. Human studies indicate that exercise performed three to five times per week, for at least 30–60 min per session, is sufficient to engage these protective mechanisms and produce measurable cognitive benefits (Dring et al., 2019). Collectively, these findings support aerobic-dominant exercise programs of moderate intensity and sufficient duration as a practical strategy to counteract ferroptosis and slow neurodegenerative progression in AD. Future studies should refine these prescriptions by integrating individual metabolic status, disease stage, and responsiveness of ferroptosis-related pathways.

**TABLE 4:** Exercise-mediated regulation of ferroptosis in AD.

Ferroptosis-related axis	Model/population	Exercise paradigm	Ferroptosis-relevant outcomes	References
Brain iron deposition	Older adults at risk of AD	Habitual physical activity	↓ hippocampal iron (QSM MRI); ↑ memory	Lee et al. (2023)
Systemic lipid peroxidation	Mild cognitive impairment	Aerobic exercise	↓ circulating lipid peroxidation markers	Ye et al. (2021)
Neuroinflammatory milieu	Mild cognitive impairment/early AD	Aerobic exercise interventions	↓ inflammatory biomarkers	Katsipis et al. (2024)
Cognitive outcome	Mild cognitive impairment	Aerobic exercise (RCTs)	↑ executive function and memory	Baker et al. (2010)
Brain structural integrity	Older adults with MCI	Aerobic exercise	↑ hippocampal volume; ↑ memory	ten Brinke et al. (2015)
Brain iron homeostasis	APP/PS1 mice	Aerobic exercise	↓ hippocampal and cortical iron; ↓ ferroptosis susceptibility	Li et al. (2024)
Lipid peroxidation pressure	APP/PS1 mice	Aerobic exercise	↓ lipid peroxidation; ↓ oxidizable Polyunsaturated fatty acids (PUFA)-enriched membranes	Ferrera et al. (2021)
GPX4-centered antioxidant defense	APP/PS1 mice	Aerobic exercise	↑ GPX4 activity; ↑ glutathione availability	Wang et al. (2024a)
Neuroinflammatory amplification	APP/PS1 mice	Aerobic exercise	↓ inflammatory signaling linked to ferroptosis	Juarez-Rendon et al. (2023)

## 5 Discussion

The recognition of ferroptosis as a central driver in AD pathogenesis marks a critical departure from amyloid-centric paradigms, offering a more integrated view of neurodegeneration. Accumulating evidence highlights cellular senescence as a key upstream event that enhances ferroptotic vulnerability by concurrently disrupting iron homeostasis, lipid metabolism, and antioxidant defenses (Masaldan et al., 2018; De Leon-Oliva et al., 2024). This senescence-induced shift creates a permissive environment for neuronal injury, where iron accumulation, peroxidation of polyunsaturated fatty acids, and collapse of the glutathione and GPX4 antioxidant axis form a triad of interconnected checkpoints that accelerate the transition from healthy aging to neurodegeneration.

Within this pathological framework, exercise emerges as a multifaceted regulator capable of intervening across several ferroptosis-prone nodes. Physical activity restores iron balance, remodels lipid profiles to reduce peroxidation susceptibility, reactivates antioxidant networks, enhances mitochondrial turnover and autophagy, and suppresses neuroinflammatory signals (Pahlavani, 2023; Hao et al., 2024; Gutierre et al., 2025). These adaptations are coordinated not only within the brain but also through peripheral organs, including liver, gut, and skeletal muscle, forming a systemic defense architecture. By targeting both the ferroptotic machinery and the senescence programs that precede it, exercise delineates a compelling therapeutic axis. Senescence increases ferroptotic sensitivity, ferroptosis accelerates AD pathology, and exercise disrupts this cascade at multiple regulatory levels.

The therapeutic potential of exercise extends well beyond the scope of conventional pharmacological strategies. Unlike single-target drugs that often fail to capture the multifactorial nature of AD, exercise acts as a pleiotropic modulator capable of synchronously targeting diverse pathological processes across organ systems (Zhang et al., 2021c; Aczel et al., 2022). This systems-level modulation is particularly effective against ferroptosis, a mechanism regulated by highly interconnected metabolic and redox pathways where compensatory loops frequently limit the efficacy of isolated interventions. Exercise exerts both preventive and restorative effects by lowering ferroptotic vulnerability in healthy neurons and promoting functional recovery in compromised cells. These benefits are reinforced through sustained adaptations, including epigenetic remodeling and metabolic memory, which extend protection beyond the active intervention period. Importantly, exercise achieves these outcomes without the adverse effects associated with iron chelators or radical scavengers, making it feasible for long-term, population-wide implementation. Its accessibility and scalability further enhance its value, especially in settings where pharmacological approaches remain inaccessible or cost-prohibitive. Critically, exercise addresses systemic contributors to brain ferroptosis, such as metabolic dysfunction, cardiovascular decline, and immune imbalance, that lie outside the reach of most central nervous system targeted therapies (Valenzuela et al., 2020; da Rocha et al., 2025). This approach offers a uniquely comprehensive strategy for modifying disease trajectory at both neural and peripheral levels.

Despite robust preclinical evidence and consistent epidemiological associations supporting exercise-induced neuroprotection, critical challenges remain in translating these findings into clinical practice. Observational studies repeatedly link regular physical activity with reduced AD risk, and early-phase intervention trials report cognitive benefits from aerobic training. However, direct validation of anti-ferroptotic mechanisms in human subjects is lacking. Most existing clinical studies do not incorporate ferroptosis-specific biomarkers, such as plasma 4-HNE, cerebrospinal fluid ferritin, or iron-sensitive neuroimaging, that could establish a causal link between exercise and ferroptosis suppression. Further complicating translation is the heterogeneity of exercise protocols across studies, including variations in type, intensity, duration, and frequency, which hinder the formulation of standardized exercise prescriptions for ferroptosis modulation. Inter-individual factors, such as APOE genotype, baseline fitness, comorbidities, and disease stage, also influence responsiveness, underscoring the need for precision exercise strategies rather than uniform recommendations. Moreover, sustaining long-term adherence remains a major barrier, particularly among older adults with mobility limitations or cognitive decline. Innovative solutions, such as socially engaging formats, virtual reality enhanced protocols, or gamified interventions, may enhance compliance. To evaluate intervention efficacy, the field urgently requires standardized tools capable of capturing both peripheral and brain-specific ferroptosis signatures.

Future research must progress along multiple complementary fronts to fully unlock the therapeutic potential of exercise in combating ferroptosis-driven neurodegeneration. At the mechanistic level, advanced platforms such as single-cell transcriptomics, spatial metabolomics, and human-derived organoid systems are essential to delineate cell type specific responses to exercise and to uncover key regulatory nodes that confer ferroptosis resistance (da Rocha et al., 2025). The development of exercise-mimetic agents that reproduce beneficial molecular adaptations could offer therapeutic alternatives for individuals with limited mobility or comorbidities precluding physical activity. For clinical translation, large-scale randomized controlled trials incorporating ferroptosis biomarkers as primary endpoints are urgently needed. Stratification by genetic risk factors, such as APOE status, and disease stage will be crucial for identifying optimal intervention windows (Martens et al., 2022). Emerging neuroimaging modalities integrating brain iron quantification, lipid peroxidation mapping, and network connectivity analyses may enable non-invasive monitoring of ferroptosis dynamics in response to exercise. Digital health technologies, including wearable sensors and smartphone-based platforms, provide opportunities for individualized dose and response monitoring and real-time modulation of exercise protocols. Multimodal strategies deserve particular focus, combining exercise with dietary modulation of iron handling, pharmacologic ferroptosis inhibitors, or cognitive training to synergistically enhance neuroprotection. Additionally, exosome-based therapies derived from exercised individuals offer a novel avenue for delivering systemic protective signals to the brain. Longitudinal cohort studies beginning in midlife will be critical to determine whether early-life exercise can delay or prevent ferroptosis-associated neurodegeneration.
